# Anti-cancer effects of DHP107 on canine mammary gland cancer examined through in-vitro and in-vivo mouse xenograft models

**DOI:** 10.1186/s12917-023-03837-4

**Published:** 2024-01-03

**Authors:** Hyung-Kyu Chae, Ye-In Oh, Ga-Hyun Lim, Yun-Chan Jung, Seol-Hee Park, Ju-Hyun An, Su-Min Park, Kyoung-Won Seo, Sung-Nam Chu, Qiang Li, Hwa-Young Youn

**Affiliations:** 1 Laboratory of Veterinary Internal Medicine, College of Veterinary Medicine, Seoul National University, Seoul, 08826 Republic of Korea; 2 Department of Veterinary Internal Medicine, Western Referral Animal Medical Center, Seoul, Republic of Korea; 3 Department of Veterinary Internal Medicine, College of Veterinary Medicine, Kyungpook National University, Daegu, Republic of Korea; 4 Laboratory Animal Center, CHA University, CHA Biocomplex, Sungnam, Republic of Korea; 5 Department of Veterinary Emergency and Critical Care Medicine and Institute of Veterinary Science, College of Veterinary Medicine, Kangwon National University, Chuncheon, Republic of Korea; 6 Pangyo Research Laboratory, DaeHwa Pharmaceutical Co. Ltd, Sungnam, Republic of Korea; 7 Department of Veterinary Medicine, College of Agriculture, YanBian University, YanJi, JiLin 133000 China

**Keywords:** Cancer, Chemotherapy, Canine, Oral paclitaxel, Mammary gland cancer

## Abstract

**Background:**

Canine mammary gland cancer (CMGC) is a common neoplasm in intact bitches. However, the benefit of adjuvant chemotherapy is unclear. The aim of this study was to investigate the anti-proliferative effects of paclitaxel on CMGC in *in-vitro* and *in-vivo* settings.

**Results:**

Paclitaxel dose-dependently inhibited viability and induced G2/M phase cell cycle arrest and apoptosis in both primary and metastatic CMGC cell lines (CIPp and CIPm). In animal experiments, the average tumour volume decreased significantly in proportion to the administered oral paclitaxel dose. By examining tumour tissue using a TUNEL assay and immunohistochemical staining with anti-CD31 as a marker of endothelial differentiation, respectively, it was confirmed that oral paclitaxel induced apoptosis and exerted an anti-angiogenetic effect in tumour tissues. Further, downregulation of cyclin D1 in tumour tissues suggested that oral paclitaxel induced cell cycle arrest in tumour tissues *in-vivo.*

**Conclusions:**

Our results suggest that paclitaxel may have anti-cancer effects on CMGC through cell cycle arrest, induction of apoptosis, and anti-angiogenesis. This study could provide a novel approach to treat CMGC.

**Supplementary Information:**

The online version contains supplementary material available at 10.1186/s12917-023-03837-4.

## Background

Canine mammary gland tumour is a common neoplasm in female dogs, with approximately 50% of cases revealed to be malignant on histopathology [1–3]. Canine mammary gland cancer (CMGC) and human breast cancer share many epidemiologic and pathologic features, including marked histologic and molecular heterogeneity [1]. In particular, certain types of CMGCs have similar histological features to human breast cancer originating mainly from epithelial cells [2, 4]. For this reason, CMGCs of epithelial origin are used as a model for human breast cancer research [1]. Although classification systems and associated targeted therapy for human breast cancer have been extensively researched, studies on adjuvant therapies for CMGC are scarce. Thus, adjuvant chemotherapy has not been demonstrated to have a clear benefit in CMGC [1, 5, 6]. The mortality rate related to CMGC within 1 year of diagnosis is over 40%, indicating the need for novel approaches to treat CMGC [7]. As a result of these needs, research on various drugs and mechanisms, other than surgical resection, have been conducted as a way to manage CMGC [1, 5, 8, 9]. In these studies, it has been demonstrated that paclitaxel induces tumour cell apoptosis on canine mammary gland cells and can be used for treatment.

Paclitaxel is a microtubule-stabilizing drug that targets tubulins, resulting in cell cycle arrest [10]. It is used to treat various human cancers, such as ovarian, breast, and non-small cell lung tumours [11–13]. However, conventional intravenous paclitaxel is associated with the adverse effect of fatal hypersensitivity reaction despite pretreatment because of the addition of Cremophor EL to solubilize insoluble paclitaxel [14, 15]. This hypersensitivity reaction has also been reported in dogs, contraindicating the use of intravenous paclitaxel in CMGC [16].

Formulations of paclitaxel without Cremophor EL have been designed to reduce the risk of hypersensitivity reaction [17, 18]. Among them, an oral paclitaxel formulation administered by weekly schedule has shown the advantages of easy administration and a long duration of action [19, 20]. DHP_107_, a novel oral paclitaxel containing monoolein, tricaprylin, and polysorbate 80, is designed to be mucoadhesive, facilitating adhesion to mucosal cells in the gastrointestinal tract [21]. It has been approved by the United States Food and Drug Administration as a treatment for gastric cancer, and its therapeutic effects have been demonstrated in several cancers in humans [20, 22, 23]. Recently, various studies have been conducted on whether paclitaxel in a new formulation can be used as a therapeutic agent for animals with various cancers [24–28]. However, its effects have not been studied in CMGC. Therefore, the aim of this study was to investigate the efficacy of chemotherapy with DHP_107_ in a xenograft mouse model of CMGC. This study could provide a novel approach to treat CMGC using DHP_107_.

## Results

### Cytotoxic effects of paclitaxel on CMGC cell proliferation

**Fig. 1:**
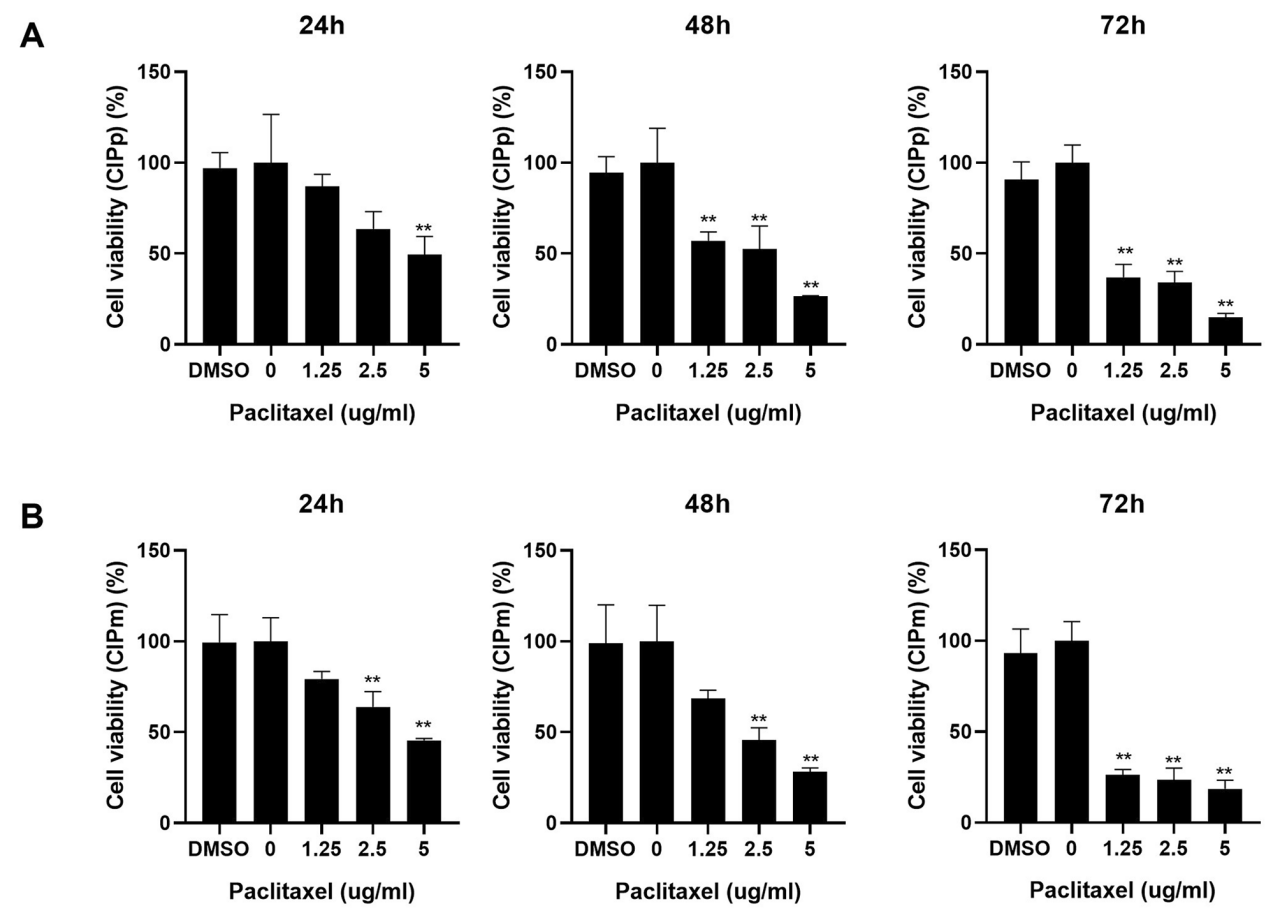
Changes in cell viability in response to oral paclitaxel in canine mammary gland tumour cell lines. (**A, B**) The viabilities of CIPp and CIPm cells treated with paclitaxel at different concentrations were analysed by CCK assay. All experiments were independently conducted in triplicate. Data are expressed as mean ± standard deviation. Statistically significant differences in relation to control are indicated by ‘*’ or ‘**’. *p < 0.05, **p < 0.01

To determine the effects of oral paclitaxel on the proliferation of CMGC cells, CCK assay was performed. The viabilities of CIPp and CIPm cells treated with paclitaxel for 24 h at concentrations of 0, 1.25, 2.5, and 5 µg/mL decreased in a dose-dependent manner (Fig. 1). There was no difference between the viability of the 0 µg/mL and 0.5% DMSO-treated cells. After 48 and 72 h of paclitaxel treatment, a greater decrease in cell viability was observed in both cell lines compared to the cell viability after 24 h (Fig. 1). These CCK assay results indicated the inhibitory ability of paclitaxel against primary CMGCs and metastatic cancer cell lines.

### Effect of paclitaxel on cell cycle arrest in CMGC cell lines

**Fig. 2:**
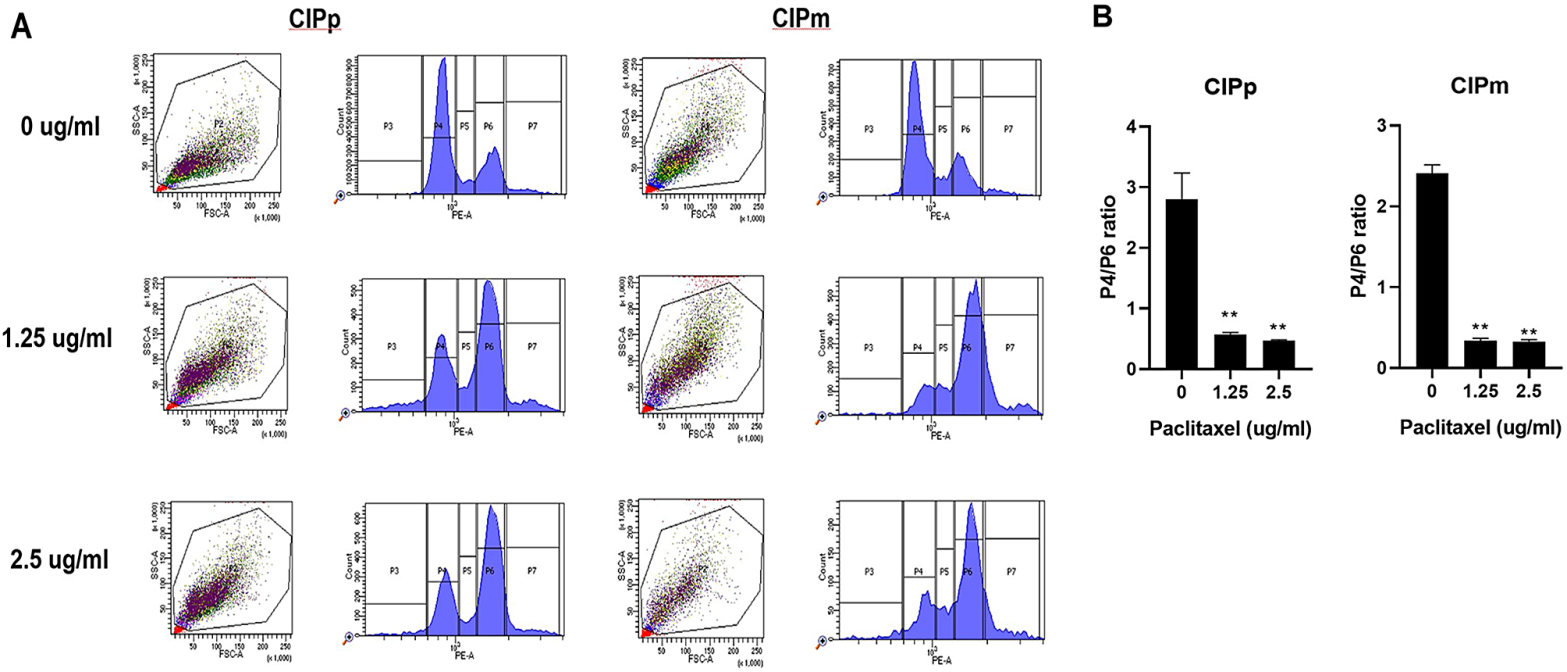
Changes in cell cycle in response to oral paclitaxel in canine mammary gland tumour cell lines. (**A**) Cell-cycle distribution of CIPp and CIPm cells treated with oral paclitaxel was analysed by flow cytometry. (**B**) P4/P6 ratio of CIPp and CIPm cells. All experiments were independently conducted in triplicate. Data are expressed as mean ± standard deviation. Statistically significant differences in relation to control are indicated by ‘**’. **p < 0.01

Flow cytometry was used to analyse the cell cycle distribution in CMGC treated with oral paclitaxel. CIPp and CIPm cells treated with paclitaxel at concentrations of 0, 1.25, and 2.5 µg/mL were analysed for the cell cycle by flow cytometry after 48 h. Cells in the G0/G1, S, and G2/M phases of the cell cycle were divided into P4, P5, and P6 categories, respectively. The cell number present in P4 categories was affected by paclitaxel treatment. In both cell lines, paclitaxel-treated cells accumulated more cells in the G2/M phase than in the control, resulting in a decreased P4/P6 ratio (Fig. 2). The P4/P6 ratio was significantly lower in paclitaxel-treated cells than in the control group. The P4/P6 ratio was lower in both cell lines treated with 2.5 µg/mL paclitaxel than in those treated with 1.25 µg/mL paclitaxel, although without statistical significance. Thus, oral paclitaxel induces G2/M phase cell cycle arrest in CMGC cell lines.

### Effect of paclitaxel on apoptosis in CMGC cell lines

**Fig. 3:**
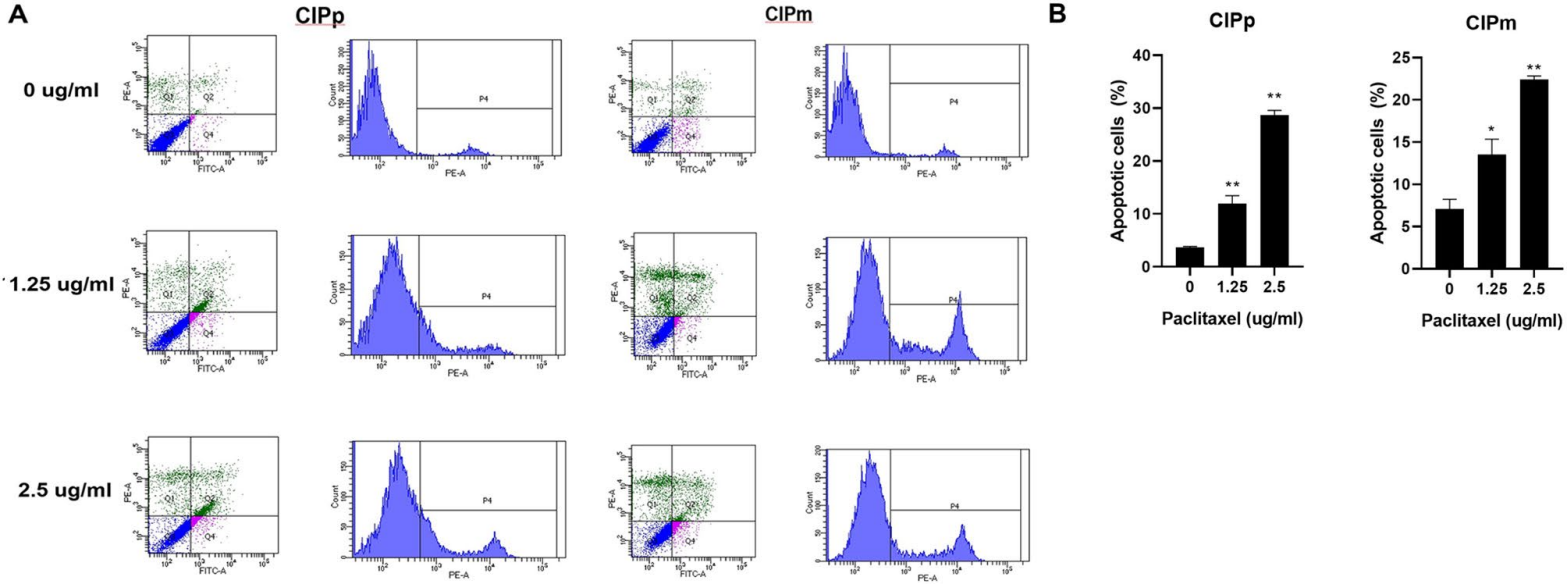
Apoptosis in canine mammary gland tumour cell line in response to oral paclitaxel. (**A**) The percentage of apoptotic cells was measured by flow cytometry. Total apoptotic cells were quantified using Annexin V (FITC) and propidium iodide (PI) double staining. (**B**) Annexin V^+^/PI^−^ cells were considered early apoptosis and Annexin V^+^/PI^+^ cells were considered late apoptosis. All experiments were independently conducted in triplicate. Data are expressed as mean ± standard deviation. Statistically significant differences in relation to control are indicated by ‘*’ or ‘**’. *p < 0.05, **p < 0.01

To evaluate apoptosis, CIPp and CIPm cells treated with paclitaxel at concentrations of 0, 1.25, and 2.5 µg/mL for 48 h were harvested and analysed by flow cytometry after Annexin V/PI dual staining. Early apoptotic cells were stained with Annexin V^+^/PI^−^, and late apoptotic cells were stained with Annexin V^+^/PI^+^. The proportion of apoptotic cells significantly increased in a dose-dependent manner compared to the control in both cell lines (Fig. 3). These results demonstrated that oral paclitaxel induced apoptosis in CMGC cell lines.

### Effect of paclitaxel on CMGC in xenograft mouse models

**Fig. 4:**
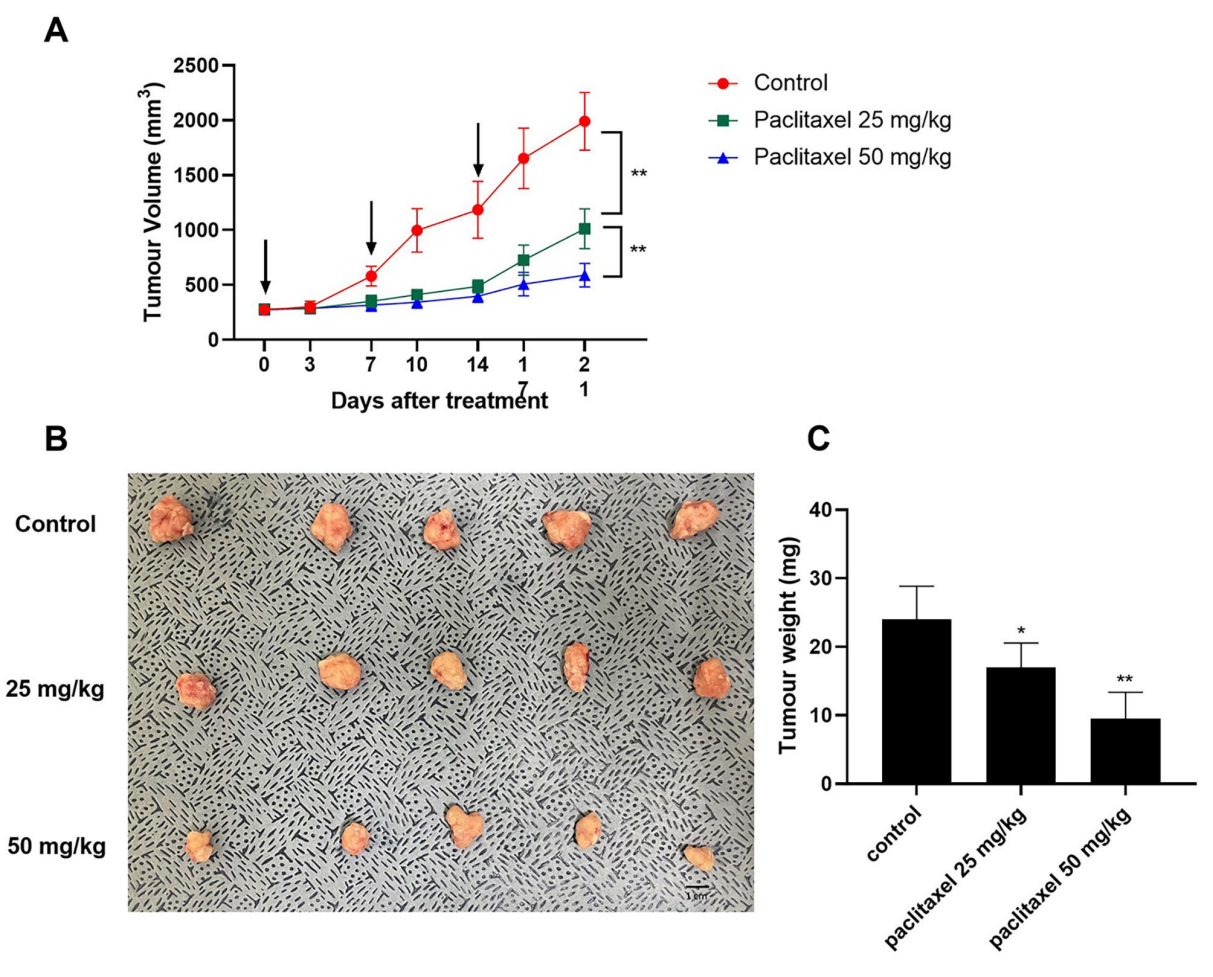
In-vivo effects of oral paclitaxel in the xenograft model. (**A**) Changes in the mean tumour volume by group during paclitaxel treatment. Paclitaxel administrations are indicated by vertical arrows. (**B**) Image of collected tumours from the xenograft models after 21 days of treatment. (**C**) Tumour weights obtained after sacrifice of mice were measured by group. Data are expressed as mean ± standard deviation. Statistically significant differences in relation to control are indicated by ‘*’ or ‘**’. *p < 0.05, **p < 0.01

To further investigate the therapeutic effect of oral paclitaxel on CMGC *in-vivo*, oral paclitaxel at concentrations of 0, 25, and 50 mg/kg every week were administered to nude mice xenografted with CIPp cells. Paclitaxel treatment was started on post-injection day 21, when the tumour size could be easily measured. The tumour size did not differ among groups at the start of treatment. However, on measuring the tumour size every 3–4 days, a relatively rapid increase was observed in the control group administered with the same volume of saline compared to the paclitaxel group (Fig. 4A). On treatment day 21, the mean volumes of the control, paclitaxel-25, and paclitaxel-50 groups were 2399.2 ± 387.9 mm^3^, 1336.8 ± 344.2 mm^3^, and 799.0 ± 188.4 mm^3^, respectively. When mice were sacrificed on treatment day 21, a significant decrease in weight was observed in the paclitaxel group compared to the control group (Fig. 4B-C).

**Fig. 5:**
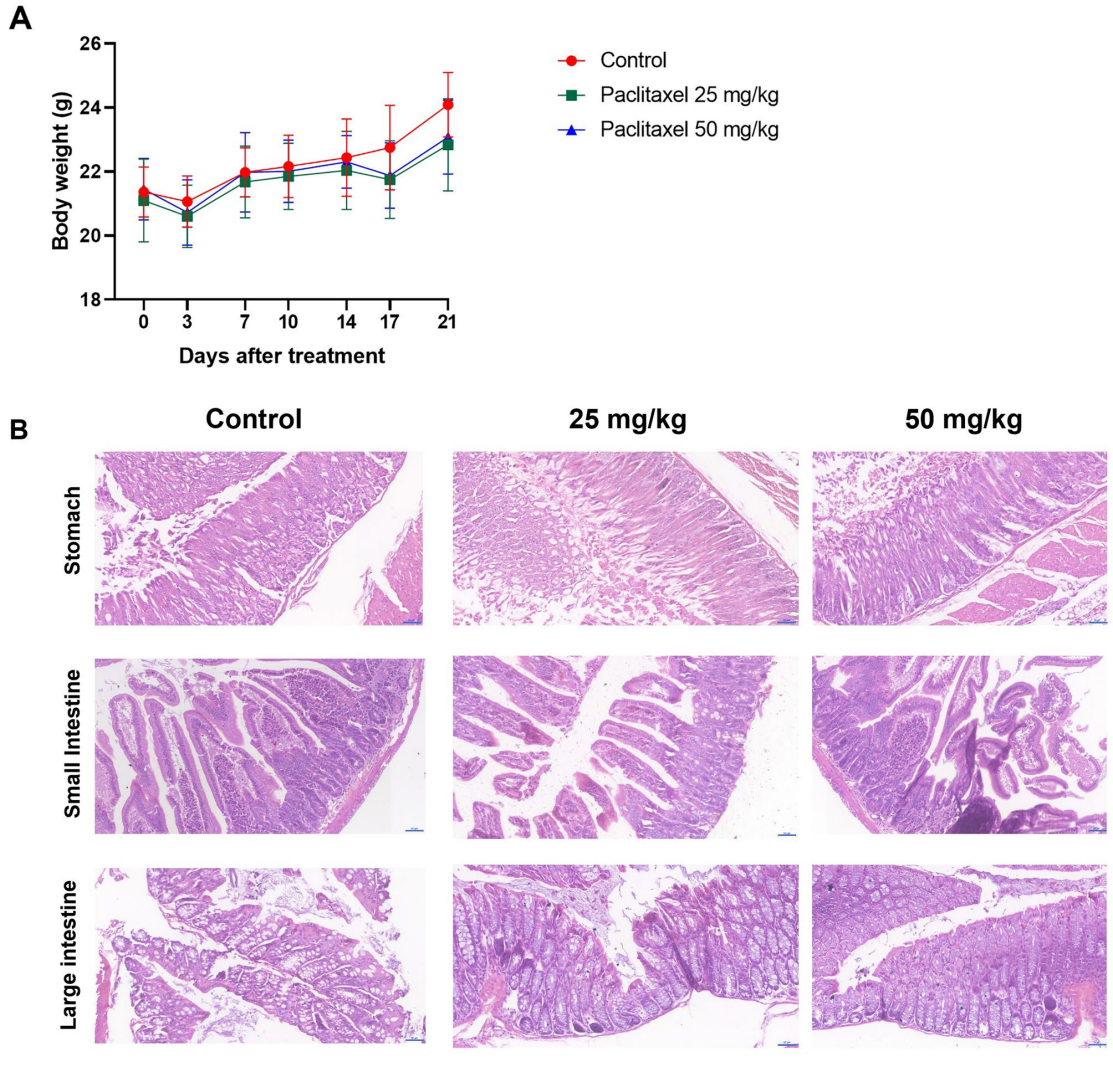
Toxicity of oral paclitaxel in the xenograft model. (**A**) The body weights of mice were monitored twice a week during paclitaxel treatment. Data are expressed as mean ± standard deviation. (**B**) After 21 days of treatment, mice were sacrificed. Gastrointestinal tissues were sampled to evaluate gastrointestinal adverse events of oral paclitaxel

To evaluate drug toxicity in mice, changes in body weight and adverse events were closely monitored during the administration period. In addition, specific findings were observed during necropsy, and gastrointestinal tract tissues were collected from each group. The body weight did not differ between the groups during the treatment period. On day 21, the body weight was lower in the treatment group than in the control group, although without statistical significance (Fig. 5A). In the histopathologic analysis of gastrointestinal tissues, no abnormal findings, such as necrosis or inflammation, were observed in the paclitaxel group (Fig. 5B).

### Paclitaxel for apoptosis and anti-angiogenesis in CMGC in-vivo

**Fig. 6:**
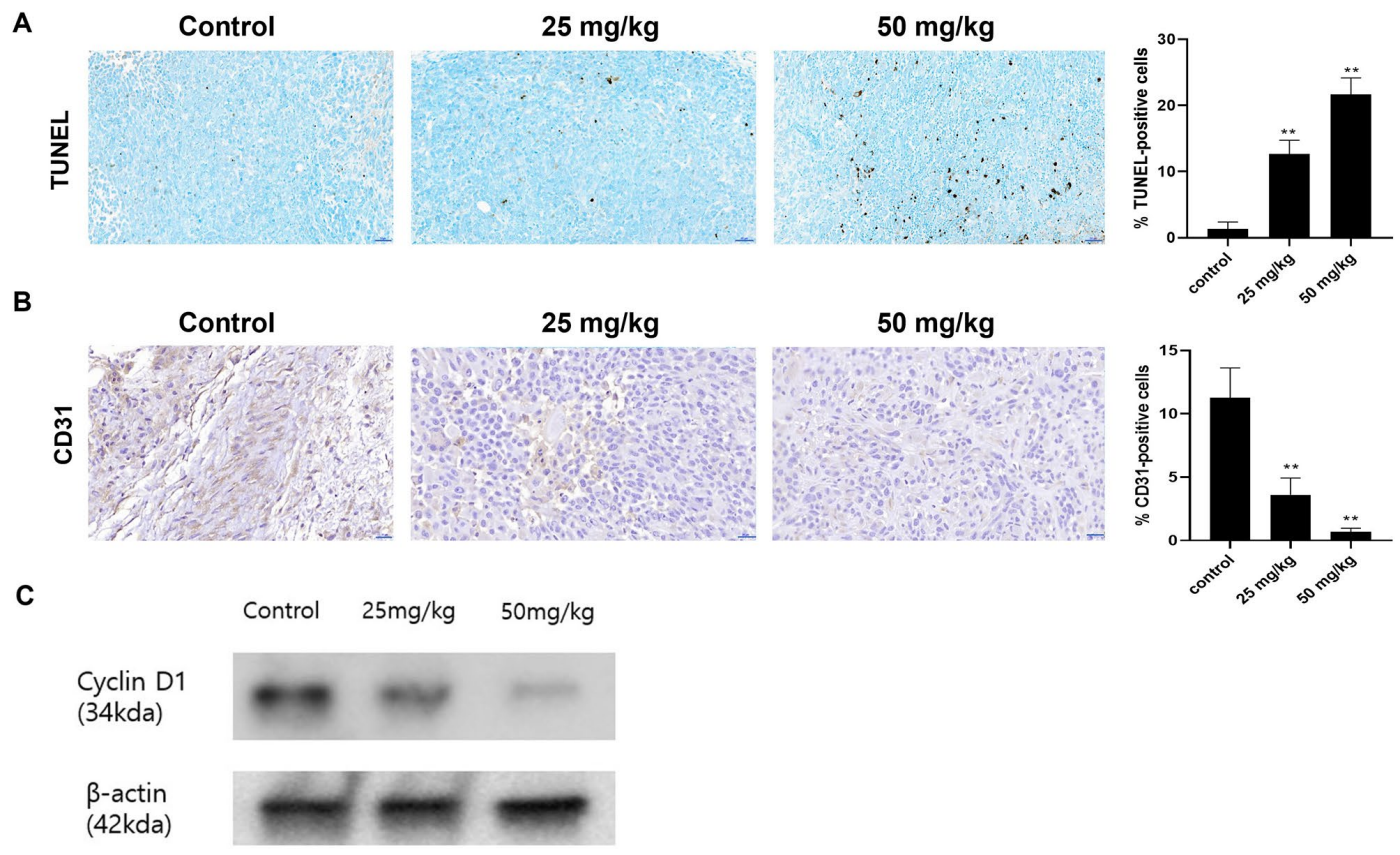
Anti-tumour activity of oral paclitaxel on tumour tissue in-vivo. (**A**) Representative images of TUNEL staining in tumour tissues obtained from the xenograft model. (**B**) Representative images of immunohistochemical staining using an anti-CD31 antibody in tumour tissues obtained from the xenograft model. Quantification of the immunohistochemistry assay was counted in six random fields per group. (**C**) The collected tumour tissues were subjected to western blot analysis for the evaluation of the expression level of cyclin-D1. Data are expressed as mean ± standard deviation. Statistically significant differences in relation to control are indicated by ‘**’. **p < 0.01

The apoptosis and anti-angiogenesis abilities of oral paclitaxel *in-vivo* were evaluated by terminal deoxynucleotidyl transferase dUTP nick-end labelling (TUNEL) assay and anti-CD31 immunochemical staining in tumour tissues. TUNEL-positive cells were significantly more in the paclitaxel group (Fig. 6A). Conversely, CD31-positive areas were smaller in the paclitaxel group, as observed by immunochemical staining for CD31 (Fig. 6B). These results demonstrated the apoptotic and antiangiogenic effects of oral paclitaxel on CMGC cells in-vivo.

### Effect of paclitaxel on cell cycle in-vivo

We further investigated the effects of oral paclitaxel on the cyclin-D1 expression by western blotting. The results revealed that cyclin-D1 expression significantly decreased in the paclitaxel group in a dose-dependent manner (Fig. 6C and Additional file 1: Supplementary Material 1). These results suggested that oral paclitaxel induced G0/G1 cell cycle arrest by downregulating cyclin-D1 in the CMGC xenograft model.

## Discussion

Although mammary gland cancers are common in female dogs, whether or not postoperative adjuvant chemotherapy prolongs the survival in CMGC remains unknown [1, 5]. There have been case reports of measurable CMGC response to paclitaxel, but postoperative chemotherapy has not been widely applied owing to the lack of efficacy studies and high hypersensitivity rate of conventional paclitaxel intravenous drugs despite pretreatment [16, 29]. It has been reported that recently developed nanosomal paclitaxel can effectively reach tumour tissues and be effective as a treatment for canine mammary neoplasms [9]. In addition, studies on substances that can increase the oral bioavailability of drugs have also been conducted [30–32]. As such, effective drug delivery to tumour tissue and increased oral bioavailability may be key strategies for successful treatment.

The recently developed oral paclitaxel (DHP107) is a safe and efficient treatment option for canine cancers based on its efficacy and safety results in various human cancers [20, 22, 23]. The paclitaxel used in this study is composed of monoolein, tricaprylin, and Tween 90, and it exerts its effect by interacting with bile acids and spontaneously forming ‘micelles’ with a diameter of approximately 10 µm in the intestine [17]. It has shown improved gastroenteric area distribution and reduced hypersensitivity compared to Taxol and been approved as a treatment for gastric cancer [17, 20, 33]. In a retrospective study, it was also suggested that DHP107 can be safely applied, without hypersensitivity, as a treatment for dogs with various cancers [27]. However, no studies have investigated the efficacy of oral paclitaxel in CMGC. Therefore, we aimed to investigate the efficacy of oral paclitaxel in CMGCs *in-vitro* and *in-vivo.*

CIPp and CIPm cells with pathological diagnostic features of carcinoma were used in this *in-vitro* study [34]. Consistent with the results of previous studies demonstrating the effectiveness of oral paclitaxel on human cancer cell lines, such as ovarian and bladder cancer cell lines, oral paclitaxel inhibited viability and induced G2/M phase cell arrest and apoptosis in both primary and metastatic CMGC cell lines [35, 36]. These results suggested that oral paclitaxel may be effective in inducing apoptosis and cell cycle arrest in primary and metastatic CMGC cell lines. In addition to the efficacy at the cell level, an animal experiment with xenograft mouse models was planned to further evaluate the effects. Our results showed that oral paclitaxel reduced tumour volume and weight without affecting body weight of CMGC-xenografted mice. In the tumour tissue analysis, decreased cyclin-D1, increased TUNEL-positive cells, and decreased CD31-positive cells were observed in the paclitaxel group. Cyclin-D1, a key regulator of the G1 phase transition during cell cycle progression, regulates the transcription of genes that promote angiogenesis and invasion [37]. Cyclin-D1 overexpression has been reported in many breast cancers, and cyclin-D1 may be a promising target for breast cancer treatment [37, 38]. The reduced cyclin-D1 expression in tumour tissues suggests that oral paclitaxel exerts a therapeutic effect by targeting cyclin-D1 in CMGC. Taken together with the results of increased cell proportions corresponding to the G2/M phase confirmed by flow cytometry, oral paclitaxel could exert a therapeutic effect by inducing both G1 and G2/M cycle arrests in CMGC. Furthermore, apoptosis and anti-angiogenesis in CMGC tissues treated with oral paclitaxel were confirmed by the TUNEL assay and CD31 immunohistochemistry staining. The aforementioned results and dose-dependent tumour size reduction without gastrointestinal abnormalities indicated that oral paclitaxel could be a safe and effective therapeutic agent for CMGCs.

This study had some limitations. First, the effectiveness of oral paclitaxel in treating all types of CMGC could not be demonstrated, since the cell lines used in this study were of a single origin. Although the CIPp and CIPm cell lines seemed to be appropriate for investigating the efficacy of oral paclitaxel in CMGCs, which mostly comprise carcinoma, further studies using other cell lines are required to determine the effectiveness of oral paclitaxel in all types of CMGCs [39, 40]. Second, the dose and route of drug delivery were limited. Drug deliveries at 25 and 50 mg/kg were set based on a previous study, but comparing various drug delivery effects may have been possible if a metronomic drug delivery schedule or intravenous drug had been added [25, 35]. Lastly, no experiments have been conducted on positive control or co-administration with drugs currently used to treat CMGCs, such as doxorubicin or mitoxantrone. These drugs are challenging to administer in metronomic manner, therefore, if additional research is conducted on the effects compared to these drugs, paclitaxel could be widely used as an alternative or in combination with these drugs.

Nevertheless, the results of this study may provide a reference for veterinary medicine regarding the anti-cancer effects of oral paclitaxel in mouse xenograft models of CMGCs. The results of this study could address the lack of an adjuvant chemotherapy protocol due to the uncertain characteristics of the effects of existing anticancer drugs on CMGC [1, 5, 41, 42]. In addition, as has recently been reported of adjuvant thalidomide metromonic chemotherapy with anti-angiogenic properties on CMGCs, oral paclitaxel is capable of dense drug delivery that may be actively considered as a therapeutic agent in dogs with mammary tumours [27, 35, 43]. We confirmed inhibition of cell viability, increased cell cycle arrest and apoptosis, and anti-angiogenic effect on tumour tissue at the cellular and *in-vivo* level by paclitaxel against CMGC through animal experiments. This suggests that this drug has the potential to have therapeutic effects in dogs with CMGCs through mechanisms we identified experimentally.

## Conclusion

Oral paclitaxel inhibits proliferation and induces apoptosis and cell cycle arrest in a concentration-dependent manner in primary and metastatic CMGC cell lines. In animal experiments, oral paclitaxel inhibited tumour growth by inducing apoptosis and exerting anti-angiogenic effects in tumour tissues. If further clinical studies in dogs with CMGCs align with the results of this study, oral paclitaxel could potentially use as a novel therapy for CMGCs.

## Methods

### Cell line validation statement and culture conditions

The CMGC cell line CIP established by N. Sasaki was used in this study [36]. The validated CIP cell lines obtained from N. Sasaki were delivered from an anonymous veterinary pharmacology laboratory. CIPp was derived from primary CMGCs, and CIPm was derived from metastatic regional lymph nodes. CIPp and CIPm cells were cultured in the Roswell Park Memorial Institute-1640 medium (Sigma-Aldrich, St. Louis, MO, USA) containing 10% foetal bovine serum (Sigma-Aldrich, St. Louis, MO, USA) and 1% penicillin and streptomycin (PAN-Biotech, Aidenbach, Germany). The cultured cells were incubated at 37 °C in a humidified atmosphere of 5% CO_2_, and the medium was replaced every 2–3 days.

### Cell proliferation assay

DHP_107_(oral paclitaxel) was provided by Daehwa Pharmaceutical Co. (Seoul, Korea). CIPp and CIPm cells were seeded in 96-well plates at a density of 2,000 cells/well. All cells were incubated with paclitaxel at concentrations ranging from 0 to 5 µg/mL. The drug was dissolved in 0.5% dimethyl sulfoxide (DMSO), and a 0.5% DMSO solution was also incubated as a control. After incubation for 24, 48, and 72 h, 10 µL of CCK solution (D-Plus™ CCK Cell Viability Assay kit; Dong-in Biotech, Seoul, Korea) was added to the cultured cells, and cell viability was analysed by measuring absorbance at 450 nm using a spectrophotometer (Bio-Rad Microplate Reader Model 680, Bio-Rad Laboratories, Hercules, CA, USA.

### Cell cycle assay

CIPp and CIPm cells were seeded in 6-well plates at a density of 2 × 10^5^ cells/well. The cells were incubated overnight at 37 °C and 5% CO_2_ and co-cultured with paclitaxel at concentrations of 0, 1.25, and 2.5 µg/mL for 48 h. Following incubation, the cells were harvested and washed with phosphate-buffered saline (PBS). The washed cells were centrifuged at 150 rcf for 3 min and then fixed with 70% ethanol at -20 °C for 2 h. Subsequently, the cells were washed again with PBS, centrifuged, and incubated with 500 µL of propidium iodide (PI)/RNase staining buffer (BD Biosciences Pharmingen, San Diego, USA) for 30 min at 25 °C. The cell cycle of the samples was analysed using fluorescence-activated cell sorting (FACS Aria II, BD Biosciences). Collected data were analysed with FACSDiva software (BD Biosciences).

### Apoptosis analysis

CIPp and CIPm cells were seeded in 6-well plates at a density of 2 × 10^5^ cells, allowed to adhere, and treated with paclitaxel at concentrations of 0, 1.25, and 2.5 µg/mL for 48 h. Subsequently, they were harvested and washed twice with PBS. Further, they were dual-stained with annexin V and PI using the Annexin V Apoptosis Detection Kit I (BD Pharmingen, San Diego, CA, USA) according to the manufacturer’s instructions. The apoptosis rate of the samples was analysed within 1 h after flow cytometry (FACS Aria II, BD Biosciences).

### Mouse xenograft model

Female athymic nude mice aged 6 weeks, weighing 19–23 g, were purchased from Nara Biotech (Seoul, Korea). All mice were housed under specific pathogen-free environmental conditions at the laboratory animal facility of the Anonymous College of Veterinary Medicine. All experimental procedures involving animals were approved by the Institutional Animal Care and Use Committee at Anonymous (approval no.: Anonymous-210825-2). To induce CMGC in mice, 5 × 10^6^ CIPp cells were suspended in 100 µL of PBS, mixed with 100 µL of Matrigel (Corning Inc., New York, USA), and injected into the mammary fat pad. Tumour growth and body weight were measured every 3–4 days until post-injection day 21. Tumour volume was estimated using the following formula: (width^2^×length)/2 [44, 45]. Mice were sacrificed on day 21 with CO_2_ asphyxiation, and xenograft tumours were harvested. For safety evaluation, organ abnormalities were visually inspected at necropsy, and the organs of digestive system (stomach, small and large intestines) were assessed by group.

### Paclitaxel treatment

Paclitaxel treatment was initiated when the tumour size was palpable around 350 mm^2^. Mice were randomly divided into three groups so that the tumour size distribution was similar (n = 8 per group): saline group, administered with 200 µL saline; paclitaxel-25 group, administered with 200 µL suspension of 25 mg/kg paclitaxel; and paclitaxel-50 group, administered with 200 µL suspension of 50 mg/kg paclitaxel every week for 3 weeks. Saline and paclitaxel suspension were delivered into the mouse stomach via an oral sonde needle (Jeungdo Bio&Plant Co., Ltd., Seoul, Korea).

### Histopathological analyses

For the histopathologic analysis of gastrointestinal tissues, the tissues were fixed in 10% formaldehyde and embedded in paraffin. For light microscopic examination, 6-µm sections were prepared and stained with haematoxylin and eosin.

### Apoptotic TUNEL assay

Apoptosis rates were detected using TUNEL staining (Apo-BrdU DNA Fragmentation Assay Kit, BioVision, San Francisco, USA) according to the manufacturer’s instructions. Briefly, sections were incubated with proteinase K for 20 min at 25°C and then incubated with hydrogen peroxide for 5 min. For the labelling reaction, the slides were covered with a labelling reaction mixture and incubated for 90 min. After stopping the reaction with a stop buffer, the tissue sections were treated for 1 h with an anti-digoxigenin peroxidase antibody, developed with 3,3’-diaminobenzidine solution, and counterstained with methyl green. TUNEL-positive cells were counted in six randomly chosen fields (×400) per group. The minimal cells count per field of view was 1,000.

### Immunohistochemical analyses

For the immunohistochemical analysis, xenograft tumour tissues were fixed in 10% formaldehyde, and 6-µm paraffin-embedded tissue sections were deparaffinized in xylene and rehydrated. To analyse angiogenesis ability, the tumour tissue slides were incubated for 2 h at 25°C with an anti-CD31 antibody (1:100, LSBio, Seattle, USA). Following incubation, the tumour sections were rinsed and incubated with goat anti-mouse IgG horseradish peroxidase-labelled secondary antibody (1:100, Thermo Fisher Scientific) for 1 h at 25°C. Staining was revealed by the 3,3’-diaminobenzidine substrate, and the slides were viewed under bright-field microscopy. The percentage of the CD31-positive area was analysed using ImageJ software (ImageJ 1.43u, National Institutes of Health, USA) with a minimum count of 1,000 cells in six randomly chosen fields (×400) per group.

### Western blotting

Proteins were extracted from frozen tissue using PRO-PREP Protein Extraction Solution (iNtRON Biotechnology, Seongnam, Korea) on ice, according to the manufacturer’s instructions. Protein concentrations were measured using a DC protein assay kit (Bio-Rad, Hercules, CA, USA). Afterwards, 20 µg of protein extracted from each line was separated using sodium dodecyl sulfate gel electrophoresis, and the separated proteins were transferred onto polyvinylidene difluoride membranes (EMD Millipore, Billerica, MA, USA). Membranes were blocked with 5% skim milk in Tris-buffered saline for 1 h and incubated with an anti-cyclin-D1 antibody (1:1000; Cell Signaling Technology, Beverly, Massachusetts, USA) at 4 °C overnight. Following incubation, the membranes were washed and incubated with a goat anti-rabbit horseradish peroxidase-labelled secondary antibody (1:5000; Bethyl Laboratories, Montgomery, TX, USA) for 1 h. Immunoreactive bands were normalized to β-actin (1:1000; Santa Cruz Biotechnology) as a loading control and visualized using ImageQuant Las 4000 mini (GE Healthcare Life Sciences, Chicago, IL, USA). Band densities were analysed using the ImageJ software (ImageJ 1.43u, National Institutes of Health, USA).

### Statistical analysis

All experimental data were analysed using GraphPad Prism v.8.0.2 software (GraphPad Software Inc., La Jolla, CA, USA). The Shapiro–Wilk test was used to perform a normality test for the data. Data are expressed as mean ± standard error. The one-way analysis of variance or Student’s *t*-test was used for between-group differences. Statistical significance was set at *p* < 0.05.

### Electronic supplementary material

Below is the link to the electronic supplementary material.

Supplementary Material 1

## Data Availability

The datasets analysed and the materials used for this study are available from the corresponding author upon reasonable request.

## Abbreviations

CMGC: Canine mammary gland cancer
DMSO: dimethyl sulfoxide
PBS: phosphate-buffered saline
FACS: fluorescence-activated cell sorting
PI: propidium iodide
TUNEL: terminal deoxynucleotidyl transferase dUTP nick-end labelling
