# Supplementary figures and images for: Anti-cancer effects of DHP107 on canine mammary gland cancer examined through in-vitro and in-vivo mouse xenograft models
Source: BMC Vet Res. 2024 Jan 3;20:3. doi: 10.1186/s12917-023-03837-4 (PMC10763473; doi:10.1186/s12917-023-03837-4)

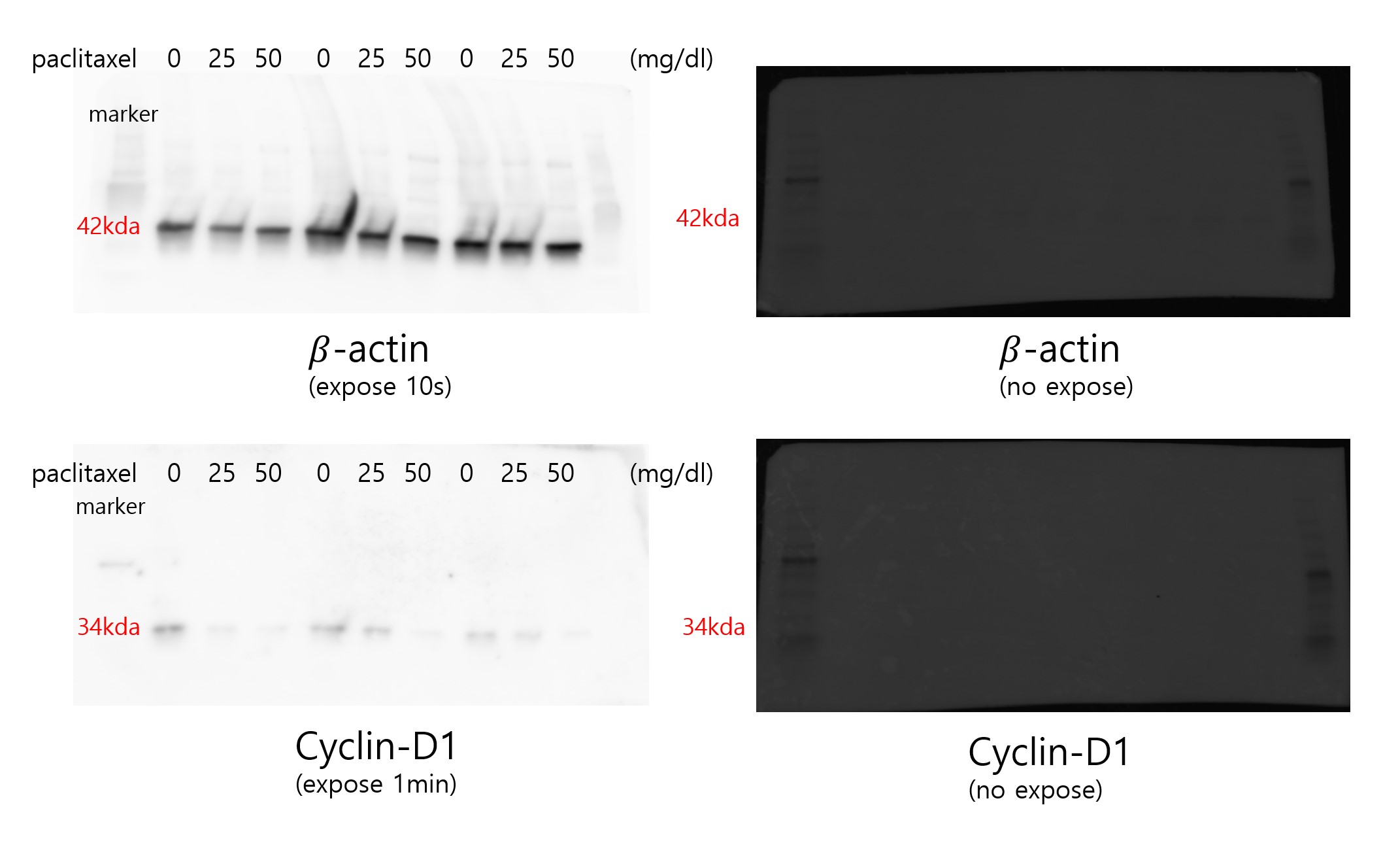

Supplement: Supplementary file 1 — Supplementary Material 1 [file 12917_2023_3837_MOESM1_ESM.jpg]
